# The Application of Magnetic-Controlled Capsule Gastroscopy in Patients Refusing C-EGD: A Single-Center 5-Year Observational Study

**DOI:** 10.1155/2021/6934594

**Published:** 2021-10-12

**Authors:** Lihan Zhou, Sijia Wang, Jian Li, Jie Zhong, Ling Zhang, Ruizhe Shen, Bielike Kouken, Chunhua Zhou, Qi Wang, Yuting Qian, Duowu Zou, Ye Chu

**Affiliations:** ^1^Department of Gastroenterology, Ruijin Hospital, Shanghai Jiao Tong University School of Medicine, Shanghai 200025, China; ^2^Department of Emergency Medicine, Zhongshan Hospital, Fudan University, Shanghai 200032, China; ^3^Clinical Research Center, Ruijin Hospital, Shanghai Jiao Tong University School of Medicine, Shanghai 200025, China

## Abstract

**Background and Aims:**

Screening for gastric diseases in symptomatic outpatients with conventional esophagogastroduodenoscopy (C-EGD) is expensive and has poor compliance. We aimed to explore the efficiency and safety of magnetic-controlled capsule gastroscopy (MCCG) in symptomatic outpatients who refused C-EGD.

**Methods:**

We performed a retrospective study of 76794 consecutive symptomatic outpatients from January 2014 to October 2019. A total of 2318 adults (F/M = 1064/1254) in the MCCG group who refused C-EGD were matched with adults in the C-EGD group using propensity-score matching (PSM). The detection rates of abnormalities were analyzed to explore the application of MCCG in symptomatic patients.

**Results:**

Our study demonstrated a prevalence of gastric ulcers (GUs) in patients with functional dyspepsia- (FD-) like symptoms of 8.14%. The detection rate of esophagitis and Barrett's esophagus was higher in patients with typical gastroesophageal reflux disease (GERD) symptoms than in patients in the other four groups (*P* < 0.01). The detection rates of gastric ulcers in the five groups (abdominal pain, bloating, heartburn, follow-up, and bleeding) were significantly different (*P* = 0.015). The total detection rate of gastric ulcers in symptomatic patients was 9.7%. A total of 7 advanced carcinomas were detected by MCCG and confirmed by endoscopic or surgical biopsy. The advanced gastric cancer detection rate was not significantly different between the MCCG group and the C-EGD matched group in terms of nonhematemesis GI bleeding (2 vs. 2, *P* = 1.00). In addition, the overall focal lesion detection rate in the MCCG group was superior to that in the C-EGD matched group (224 vs. 184, *P* = 0.038). MCCG gained a clinically meaningful small bowel diagnostic yield of 54.8% (17/31) out of 31 cases of suspected small bowel bleeding. No patient reported capsule retention at the two-week follow-up.

**Conclusion:**

MCCG is well tolerated, safe, and technically feasible and has a considerable diagnostic yield. The overall gastric diagnostic yield of gastric focal lesions with MCCG was comparable to that with C-EGD. MCCG offered a supplementary diagnosis in patients who had a previously undiagnostic C-EGD, indicating that MCCG could play an important role in the routine monitoring and follow-up of outpatient. MCCG shows its safety and efficiency in symptomatic outpatient applications.

## 1. Introduction

Gastric problems, such as peptic ulcers, polyps, and gastric cancer, are common in daily clinical practice. At least 20% of the population has chronic symptoms that can be attributed to disorders of gastroduodenal function [1], and in order to diagnose functional dyspepsia (FD) or irritable bowel syndrome (IBS), upper endoscopy is necessary to rule out structural disease [2]. Although the optimal age cut-off for endoscopic evaluation in patients with dyspepsia is controversial, gastric cancer is the second leading cause of cancer-related deaths in China [3]. Many patients are inoperable at the time of the final diagnosis of gastric cancer. The outcomes of gastric adenocarcinoma with distant metastasis are poor, with a median survival of approximately 1 year [4]. Conventional esophagogastroduodenoscopy (C-EGD) and endoscopic biopsy are the gold standards for the diagnosis of gastric diseases. The application of C-EGD for mass screening in symptomatic outpatients is limited due to the shortage of well-trained endoscopists and poor acceptance across populations [5].

The wide use of anesthesia can improve patient acceptance, but the risks of adverse events and contraindications in certain groups of patients are higher [6]. On the other hand, with proper bowel preparation, the examination of the upper gastrointestinal (GI) tract, small bowel, and part of the large intestine can be performed safely and comfortably using a single magnetic-controlled capsule gastroscopy (MCCG) procedure rather than a separate C-EGD and small-bowel capsule gastroscopy in chosen groups [7]. Therefore, painless MCCG provides a novel and promising approach for screening gastric and small bowel diseases.

Most previous studies have focused on the application of MCCG in specific groups, such as elderly [8], minor [9], recurrent and refractory iron deficiency anemia (IDA) [7], acute upper GI bleeding [10], and asymptomatic individuals [11]. The diagnostic accuracy of MCCG has previously been shown to be comparable with that of gastroscopy [12, 13]. In this paper, we intend to study how MCCG benefits symptomatic patients with more real-world enrolled samples.

## 2. Materials and Methods

### 2.1. Study Design

We performed a single-center 5-year retrospective study in outpatients from Ruijin Hospital. A total of 78075 outpatients (76794 adults and 1281 children) who had indications for endoscopy with ongoing concerns were enrolled in this study from January 2014 to October 2019 at the Department of Gastroenterology in Ruijin Hospital, Shanghai Jiao Tong University School of Medicine. Within the study period, 74476 adults underwent conventional C-EGD as first recommended by doctors. Additionally, 2318 adults who refused C-EGD underwent MCCG. The majority of reasons for refusing C-EGD could be divided into subjective and objective reasons: subjective reasons included patients' fear of discomfort caused by intubation and the procedure as well as unwillingness to undergo anesthesia; objective reasons included conventional EGD- and anesthesia-related contraindications, such as cardiopulmonary codisease, OSAS, and trismus. The exclusion criteria for MCCG included implantation of permanent pacemakers or other magnetically or electrically controlled devices, pregnancy, and patients at high risk of capsule retention, such as dysphagia. Written informed consent was obtained from all individual participants included in the study. Our study was endorsed by the Ethics Committee of Ruijin Hospital.

**Figure 1:**
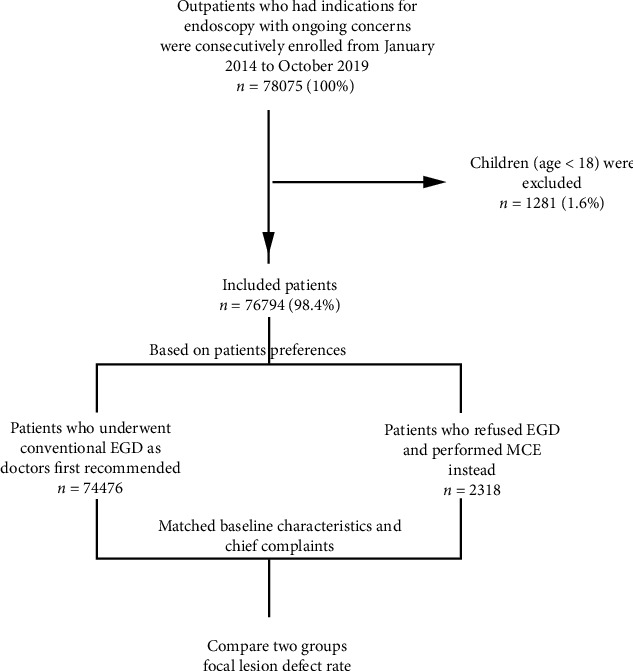
Study flowchart. ^∗^Children aged less than 18 years were enrolled in another MCCG study cohort in our center.

After data collection, we additionally compared baseline and matched characteristics (age and sex) and main complaints (abdominal bloating, abdominal pain, typical reflux symptoms, and nonhematemesis GI bleeding) using the propensity-score matching method (Figure 1). The matched tolerance was set as 0-0.02 based on the subgroup sample size.

### 2.2. Data Collection

Data collected retrospectively included sex, age, chief complaints, history of present illness, family history, surgery history, whether C-EGD had been performed previously, and whether the patient had HP infection or alcohol addiction.

Pathology was documented and double reviewed by two senior doctors. Endoscopists who had performed <2000 C-EGDs were excluded. Based on endoscopic findings, an opinion was recorded by the endoscopist as to whether patients needed further invasive investigation. Outpatients without positive findings were discharged for other outpatient evaluations.

### 2.3. Equipment

The NaviCam™ magnetic-guided capsule gastroscopy system (Shanghai ANKON Medical Technology Co. Ltd.) was applied in this study. The components included a capsule robot, magnetic-guided capsule gastroscopy examination bed, translation rotary table, magnet, console, portable recorder, capsule locator, and ESNavi software. The capsule robot consisted of capsule-like equipment with a size of 12 mm × 28 mm that took 2 frames per second. The observation view was 140 ± 10°; the working temperature was 20–40°C, and the working time was >8 h. The captured data of the capsule could be instantly transmitted by the data line to the operating table for real-time observation. The activity of the capsule was controlled by the C-arm magnetic field system. The patients in the matched C-EGD group underwent standard endoscopy (Olympus, Fukushima, Japan) without anesthesia.

### 2.4. Preparations

In patients with suspected small bowel diseases, 2000 mL of polyethylene glycol (PEG) solution was taken the night before the examination for bowel preparation, and the patients fasted overnight. On the day of the examination, the patients were asked to take 200–300 mL of water mixed with 10 mL of simethicone (Bo Xi, Berlin-Chemie AG) 60 min before the examination [14–16]. All metal belongings were removed (keys, metal dentures, mobile phones, watches, magnetic cards, etc.). During the inspection process, if the vision was not clear, patients were asked to continue drinking water until the field of view was satisfactory [17].

### 2.5. Statistics

Advice was sought from the Statistics Resource Centre of Ruijin Hospital. Visual methods (histograms and QQ Plots) and normal test methods (the Kolmogorov-Smirnov test) were used for the normality test. Parameters with a normal distribution were expressed as the mean and the standard deviation and were analyzed using Student's *t*-test. Parameters that did not have a normal distribution were expressed as the median and the interquartile range and were analyzed using the Mann–Whitney test. The chi-square test or Fisher's exact test was used to compare proportions. *P* < 0.05 was considered statistically significant in all analyses. Student's *t*-test and PSM (propensity score matching) methods were performed using SPSS 24.0 (SPSS Inc., Chicago, Illinois, USA). A fixed-effects model was conducted for the subgroup analysis, and the forest plot drawing was finished by R (version 3.6.1).

## 3. Results

The study cohort included 2318 patients refusing C-EGD with ongoing clinical concerns (male/female = 1064 : 1254 = 1 : 1.18) who underwent MCCG examination (Table 1).

The chief complaints of outpatients who refused C-EGD were generally categorized into 5 groups: (1) abdominal bloating (*n* = 1399); (2) abdominal pain without evidence of GI bleeding or other organ diseases (*n* = 542); (3) typical reflux symptoms (*n* = 144) such as heartburn and/or regurgitation; (4) nonhematemesis GI bleeding (*n* = 78), including a positive fecal occult blood test with IDA (*n* = 37), melena (*n* = 31), and hematochezia (*n* = 10); and (5) regular reexamination after standard treatment (*n* = 155), including new onset symptoms with a history of chronic gastritis (*n* = 81), reexamination after standard PPI treatment for peptic ulcers (*n* = 35), HP eradication for HP gastritis (*n* = 20), follow-ups after endoscopic treatments (*n* = 15), and postoperative radio-chemotherapy for head and neck tumor GI metastasis (*n* = 4).

**Figure 2:**
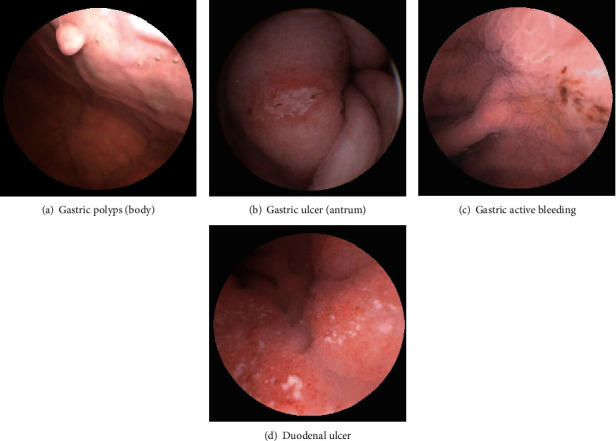
Representative focal (a–d) lesions detected by MCCG.

**Figure 3:**
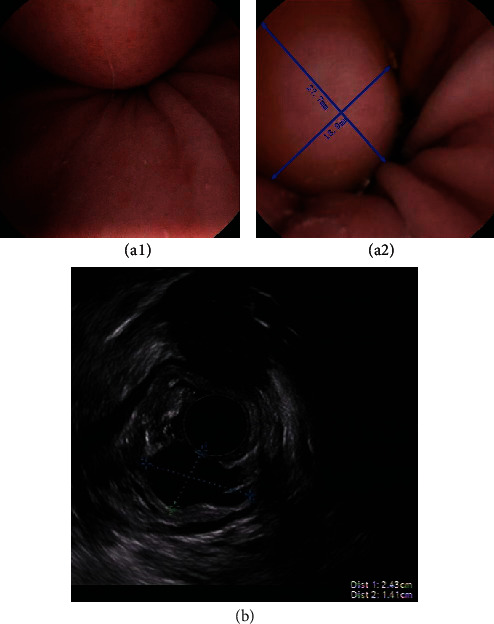
A case of SMT identified by MCCG (a) and confirmed with EUS (b).

The results of MCCG in terms of the different chief complaints are shown in Table 2. The detection rate of esophagitis was higher in patients with typical GERD symptoms, such as acid reflux and heartburn, than in the other four groups: abdominal pain, bloating, follow-up, and bleeding (11.8% vs. 0.2%, 0.6%, 0%, 0%, respectively, *P* < 0.01). Similarly, the detection rate of Barrett's esophagus was higher in patients with typical GERD symptoms than in patients with abdominal pain, bloating, follow-up, and bleeding (4.9% vs. 0.4%, 0.4%, 0.6%, 0%, respectively, *P* < 0.01). The detection rates of gastric ulcers in the five groups of abdominal pain, bloating, heartburn, follow-up, and bleeding were significantly different (12%, 8.5%, 7.6%, 9.7%. 17.9%, *P* = 0.015, *x*^2^ = 12.325). The total detection rate of gastric ulcers in symptomatic patients was 9.7%. The total detection rate of focal ulcers and erosion lesions in symptomatic subjects by MCCG in our center was 18.9% (438/2318), 45.8% (218/438) lesions were located in the gastric antrum, 17.9% (85/438) detected lesions were located in the gastric body and fundus, and 6.3% (30/438) detected lesions were located in the duodenum. Our study demonstrated a prevalence of gastric ulcers in visits with FD-like symptoms of 8.14%. Some typical focal and extensive lesions found through MCCG are shown in Figure 2. The submucosal tumor (SMT) detection rate of symptomatic subjects by MCCG in our center was 1.0% (24/2318). Out of 24 cases of SMT, 20 were diagnosed with GIST, and 2 were diagnosed with pancreatic rest (also known as ectopic pancreas, aberrant pancreas, and heterotopic pancreas). One was diagnosed with gastric duplication cyst. These cases were all confirmed by EUS/ESD. One adult case of representative SMT who was diagnosed with gastric duplication cyst is shown in Figure 3. A total of 4 cases of gastric adenocarcinoma, 2 cases of small intestinal lymphoma, and 1 case of esophageal squamous cell carcinoma were detected by MCCG and confirmed by endoscopic or surgical biopsy.

The measurement parameters of SMT were close by two modalities. The measurement with MCCG was 27.7 mm × 18.9 mm and was 24.3 mm × 21.4 mm with EUS. The patient was diagnosed with gastric duplication cyst by surgical pathology.

**Figure 4:**
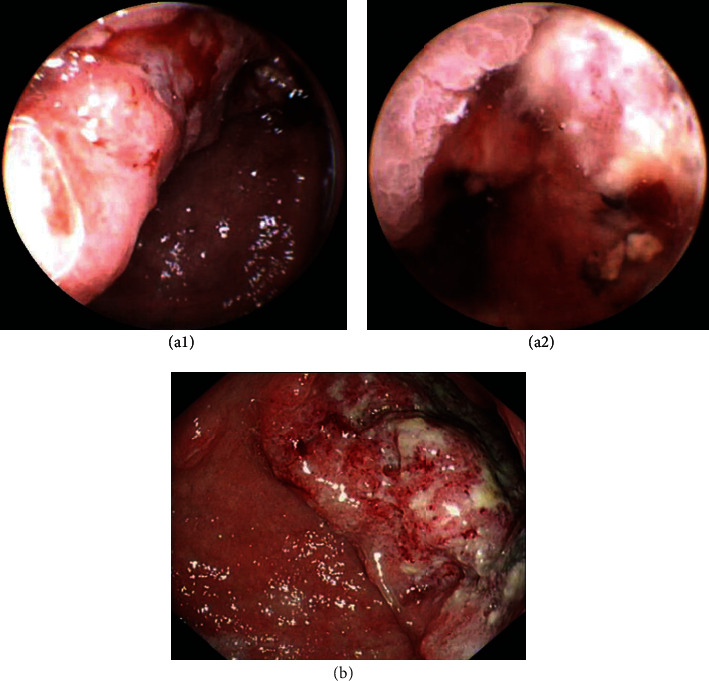
Poorly differentiated adenocarcinoma on the angle of the stomach identified by MCCG (a) and confirmed with biopsy from following C-EGD (b).

The results of MCCG in terms of nonhematemesis GI bleeding are shown in Table 3. A total of 78 patients with nonhematemesis gastrointestinal bleeding were included in the analysis, of whom 10.3% (8/78) had a history of taking antiplatelet drugs or nonsteroidal anti-inflammatory drugs (NSAIDs). MCCG gained a clinically meaningful small bowel diagnostic yield of 54.8% (17/31) out of 31 cases of suspected small bowel bleeding (recent negative C-EGD and colonoscopy). Two cases of poorly differentiated adenocarcinoma were found through MCCG and confirmed with the biopsies from the following C-EGDs (Figure 4). Both patients were on antiplatelet drugs and suffered from symptomatic IDA.

**Figure 5:**
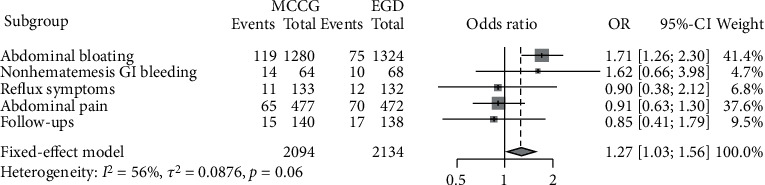
Forest plot of the comparison of different chief complaints and gastric ulcer detection rates between the MCCG and C-EGD groups. ^∗^OR: odds ratio.

The overall gastric ulcer detection rate in the MCCG group was higher than that in the C-EGD matched group (224 vs. 184, *P* = 0.038) (Table 4). The comparison of different chief complaints and gastric ulcer detection rates between the MCCG and PSM matched C-EGD groups was presented with a forest plot (Figure 5). The summary odds ratio was 1.27 (95% CI 1·03–1·56).

## 4. Safety Assessments

The gastric-intestinal preparation and the examination of MCCG were generally tolerated by all enrolled patients. All patients swallowed the capsule easily, and the examination was successfully performed in enrolled patients. Four patients experienced capsule retention in the duodenum until the battery was finished, indicating potential underlying anatomic or functional abnormalities. All patients were followed for two weeks to record adverse events and to confirm capsule excretion. Four patients who experienced temporary capsule retention were confirmed to have capsule excretion with conservative therapy. No patient reported capsule retention at the two-week follow-up.

## 5. Discussion

### 5.1. Principal Findings

The results from this study suggest that MCCG is well tolerated, safe, and technically feasible and has a considerable diagnostic yield. The overall gastric diagnostic yield of gastric ulcers with MCCG was comparable to that with C-EGD and was even better in specific groups. In those who had previously undergone C-EGD, MCCG offered a supplementary diagnosis. Even in patients who experienced capsule blockage, potential underlying anatomic or functional abnormalities were suspected, providing additional diagnostic clues for further examination. No procedure-related major adverse events were encountered in this large-sample retrospective study.

### 5.2. Advantages and Disadvantages of MCCG

Previous studies have shown that both modalities can miss lesions that are caught by the other [7, 10, 18]. Our study comparing MCCG and C-EGD in a similar population (one center, PSM patients' characteristics) would be helpful in clarifying the roles of each modality. MCCG detected more gastric ulcers than C-EGD overall, especially in patients with abdominal bloating and nonhematemesis GI bleeding. One possible explanation is that MCCG observes the stomach in its natural status, whereas C-EGD cannot reach the stomach only if the operator overinflates the cavity. Additionally, MCCG may achieve a closer view of every inch of the gastric mucosa, which makes minimal lesions easier to detect than with C-EGD. A capsule endoscope may emphasize small and tiny lesions, so we would like it to be a screening tool in the future of outpatient management since MCCG has ideal sensitivity [12]. Hence, our purpose was not to replace the role of C-EGD in high-risk patients but to explore the efficiency and supplementary practical application of MCCG in patients refusing C-EGD.

We found two cases of poorly differentiated adenocarcinoma through MCCG; these patients were on antiplatelet drugs and suffered from symptomatic IDA. In daily medical practice, GI bleeding caused by antiplatelet drugs might overlap with alarm symptoms of gastric carcinoma, resulting in the delay of diagnosis when patients show poor compliance towards conventional C-EGD. Consequently, doctors tend to take a more conservative approach when dealing with patients on antiplatelet drugs or anticoagulants [19], despite the guidelines recommended that there is no need to suspend antiplatelet or anticoagulation therapy for C-EGD with or without a biopsy [20–22]. A prospective, single-center study using WCE (wireless capsule endoscopy) and colonoscopy assessed the source of gastrointestinal bleeding for C-EGD-negative NSAID or acetylsalicylic acid (ASA) users, and 27.5% of NSAID and 19.1% of ASA users as well as 4.5% of patients without a suspected medication history were found to have small bowel bleeding (NSAID vs. non-NSAID users *P* = 0.007; ASA vs. non-ASA users *P* = 0.033), suggesting that small bowel bleeding may have been underestimated [23]. Under such conditions, MCCG shows its superiority: well-tolerated, better patient compliance, no bleeding risk due to the procedures, and no need to suspend antiplatelet or anticoagulation therapy, expand the horizon to the small bowel or even further to the large intestine when proper bowel preparation is achieved. These advantages are particularly significant in patients with cardiopulmonary codisease because the pain or discomfort of intubation might worsen hypoxia or increase the workload of the heart [6, 24]. Anesthesia is also not an optimal option for such patients because most of the anesthesia drugs frequently used, such as propofol, have cardiac or pulmonary depressive effects [6, 25, 26]. The study of Qian et al. supports that MCCG offers considerable benefit and is generally safe for elderly patients with severe angiocardiopathy or respiratory disorders [8]. MCCG may be used as an initial screening tool in patients with a history of ADA or NSAID use and gastrointestinal bleeding. Moreover, previous research on the application of MCCG in IDA patients suggests that MCCG as a preliminary screening method can reduce the overall examination time and total medical expenditure [7].

### 5.3. Implications for Clinical Practice

China has a high prevalence of gastric cancer. Our study showed that compared with the matched C-EGD group, in the MCCG group, the gastric carcinoma detection rate in patients with nonhematemesis GI tract bleeding was not significantly different. Studies of MCCG application for nonhematemesis bleeding have already been performed and have shown its advantages [7, 27]. Further investigations in large samples are required to confirm the safety and effectiveness of MCCG application in nonhematemesis bleeding. A cost-effectiveness analysis found that Chinese men and women over 50 years of age who were at high risk of gastric cancer (standardized incidence of gastric cancer is 25.9 cases per 100,000 people) and underwent upper gastrointestinal endoscopy every 2 years to screen for gastric cancer were highly in line with the cost-effectiveness principle ^28^. Moreover, 100% (4/4) of advanced malignant gastric tumors found by MCCG in our study were in patients over 50 years old, suggesting that screening with MCCG for symptomatic patients over 50 years of age is an ideal alternative for patients who have C-EGD contraindications or who refuse C-EGD.

We also investigated rebleeding patients who had recently undergone a negative C-EGD and negative colonoscopy. MCCG offered a supplementary diagnosis in 54.8% of the cases, within which the majority of pathologies were in the small bowel—indicating that MCCG could play a complementary role in the management of GI bleeding in outpatients. The diagnostic yield of MCCG in the small intestine was close to previous C-EGD studies (40%-60%). One explanation is that MCCG has better patient compliance; therefore, the earlier timing of MCCG could improve diagnostic yield ^29, 30^. Additionally, the magnetic steering of MCCG could improve the small bowel completion rate ^31^.

In patients with head and neck tumors, surgical dissection and postoperative radio-chemotherapy can cause limited mouth opening, immersive oral ulcers, and laryngeal mucosal hyperemia, making it difficult for patients to tolerate conventional C-EGD procedures. Furthermore, many chemotherapeutic drugs can cause mucosal damage, leading to digestive symptoms. A noninvasive visual tool will hopefully help rule out GI tumor metastasis, providing critical information for further medical decisions.

The prevalence of FD ranges from 5% to 11% worldwide ^32^. According to the Rome IV criteria, the diagnosis of functional dyspepsia requires upper endoscopy to rule out structural disease [2]. Postprandial abdominal bloating, early satiety, and epigastric pain/burning were the common chief complaints as the reason for outpatient visits in our study. Our study demonstrated a prevalence of gastric ulcers (GU) in visits with FD-like symptoms of 8.14%, similar to a previous study reporting a prevalence of 8.00% ^33^. Routine biopsies of duodenal ulcers (DU) with benign manifestations are not recommended, as they are unlikely to be malignant; whether a biopsy should be performed on benign-like manifested gastric ulcers is controversial and should be decided on the basis of the individual risk. For patients who refuse C-EGD in our center, patients with benign gastric and duodenal ulcers are recommended to follow a standard PPI treatment before reviewing MCCG and then perform further C-EGD examination if ulcers do not heal. The total detection rate of focal ulcers and erosion lesions in symptomatic subjects by MCCG in our center was 18.9% (438/2318); in which 45.8% (218/438) were located in the gastric antrum, 17.9% (85/438) of the detected lesions were located in the body and bottom of the stomach, and 6.3% (30/438) of the detected lesions were located in the duodenum, in line with previous reports of a trend in the transformation of peptic ulcers from GUs to DUs in the Asian population ^34^.

GIST (gastrointestinal stromal tumor) commonly occurs in the stomach and proximal small intestine but can occur in any part of the digestive tract ^35-39^. Biopsy data suggest that the incidence of subcentimeter gastric GIST lesions may be much higher than previously estimated ^36, 38^. At present, all GISTs are considered to have malignant potential, so the clinical focus is on early identification and detection and is based on the risk of recurrence and metastasis to guide further diagnosis and treatment or monitoring. However, GIST manifestations are nonspecific and include abdominal bloating, early satiety, and postprandial fullness, which are easily ignored by patients ^36, 38^. The GIST detection rate of MCCG in symptomatic subjects by our center was 0.9% (20/2318), of which 80% (16/20) came from subjects who had only abdominal distension or abdominal discomfort, indicating that MCCG can serve as a screening tool for symptomatic patients who refuse C-EGD. In particular, MCCG can be observed in the gastric cavity in its physiological state, which may facilitate the detection of SMT.

## 6. Limitation

We are facing atypical visitors with ambiguous chief complaints and different physical conditions. The subgroups were divided based mainly on the chief complaints at their first visit. Some of the complaints of visitors may vary, and there could be overlapping symptoms between groups. Since outpatients were recommended to undergo C-EGD first, those who refused C-EGD were included in the MCCG group. The MCCG group may have had patients with worse compliance or worse physical conditions, which are factors that are naturally different between MCCG and matched C-EGD patients in the PSM-based subgroup analysis.

How well MCCG could change patients' compliance was not studied. Many highly suspicious proliferative lesions were not confirmed by biopsy, because some of the patients enrolled in the MCCG group had C-EGD-related contradictions and were not eligible for further surgical operations. The median age in this study was only 38 years, and these young patients may be “worried well” and self-elected to undergo MCCG. These two factors may contribute to a relatively low carcinoma detection rate compared with other studies [11].

## Figures and Tables

**Table 1:** Clinical characteristics.

	MCCG	Matched C-EGD	*P*
Total	2318	2318	1.00
Sex (M/F)	1064/1254	1064/1254	1.00
Age (years)^#^	38 (19~94)	38 (19~94)	0.854
Reasons for visit			
Abdominal bloating	1399	1399	1.00
Abdominal pain	542	542	1.00
Reflux symptoms	144	144	1.00
Nonhematemesis GI bleeding	78	78	1.00
Follow-up after treatment	155	155	1.00

^#^Present with median (min ~ max). MCCG: magnetic-controlled capsule gastroscopy; C-EGD: conventional esophagogastroduodenoscopy.

**Table 2:** Results of MCCG in different groups.

	Abdominal pain (*n* = 542)	Abdominal bloating (*n* = 1399)	Reflux symptoms (*n* = 144)	Follow-ups (*n* = 155)	Bleeding (*n* = 78)
Sex (M/F)	214/328	663/736	63/81	78/77	42/36
Age (years)#	38 (19 ~ 81)^#^	42 (19 ~94)^#^	41 (24 ~ 80)^#^	46 (24 ~ 87)^#^	52 (25 ~ 87)^#^
Gastric ulcer	65	119	11	15	14
Duodenal ulcer	14	31	0	4	3
Jejuno-ileal ulcer	24	20	0	9	7
Esophagitis	1	8	17	0	0
Barrett's esophagus	2	6	7	1	0
Gastric SMT	4	18	0	1	1
SBST	0	1	0	0	1
Gastric polyp (antrum/fundus or body)	3/14	40/63	1/4	4/7	0/2
Duodenal polyp	0	6	2	1	0
Jejuno-ileal polyp	1	11	0	1	2
Suspected lesion^∗^	8	41	3	6	4

^#^Present with median (min ~ max). ^∗^Suspicious morphology, patients are referred to undergo C-EGD or EUS to further confirm the diagnosis. MCCG: magnetic-controlled capsule gastroscopy; C-EGD: conventional esophagogastroduodenoscopy; SMT: submucosal tumor; SBST: small bowel stromal tumors.

**Table 3:** Results of MCCG for nonhematemesis GI bleeding.

	FOBT(+)IDA	Melena	Hematochezia
	(*n* = 37)	(*n* = 31)	(*n* = 10)
Gastric ulcer	7	7	0
Duodenal ulcer	2	1	0
Jejuno-ileal ulcer	3	3	1
GIA	3	4	1
SBST	1	1	1
Parasite	0	1	0

MCCG: magnetic-controlled capsule gastroscopy; C-EGD: conventional esophagogastroduodenoscopy; SBST: small bowel stromal tumors; GIA: GI angiectasia.

**Table 4:** Comparison of gastric ulcer detection rates between the MCCG and matched C-EGD groups.

	MCCG		C-EGD		*P*
	Positive	Total	Positive	Total	
All patients	224 (9.66%)	2318	184 (7.94%)	2318	0.038
Abdominal bloating	119 (8.50%)	1399	75 (5.36%)	1399	0.001
Nonhematemesis GI bleeding	14 (17.9%)	78	10 (12.82%)	78	0.375
Reflux symptoms	11 (7.64%)	144	12 (8.33%)	144	0.828
Abdominal pain	65 (11.99%)	542	70 (11.07%)	542	0.399
Follow-ups	15 (9.68%)	155	17 (10.97%)	155	0.854

MCCG: magnetic-controlled capsule gastroscopy; C-EGD: conventional esophagogastroduodenoscopy.

## Data Availability

The data used to support the findings of this study are restricted by the Ethics Committee of Ruijin Hospital in order to protect patient privacy. Data are available from Dr. Zhou Lihan for researchers who meet the criteria for access to confidential data.

## References

[1] Stanghellini V., Chan F. K. L., Hasler W. L. (2016). Gastroduodenal disorders. *Gastroenterology*.

[2] Drossman D. A. (2016). Functional gastrointestinal disorders: history, pathophysiology, clinical features and Rome IV. *Gastroenterology*.

[3] Chen W., Zheng R., Baade P. D. (2016). Cancer statistics in China, 2015. *CA: a Cancer Journal for Clinicians*.

[4] Van Cutsem E., Sagaert X., Topal B., Haustermans K., Prenen H. (2016). Gastric cancer. *Lancet*.

[5] Early D. S., Lightdale J. R., Vargo J. J. (2018). Guidelines for sedation and anesthesia in GI endoscopy. *Gastrointestinal Endoscopy*.

[6] Ching H. L., Hale M. F., Kurien M. (2019). Diagnostic yield of magnetically assisted capsule endoscopy versus gastroscopy in recurrent and refractory iron deficiency anemia. *Endoscopy*.

[7] Zhang S., Sun T., Xie Y. (2019). Clinical efficiency and safety of magnetic-controlled capsule endoscopy for gastric diseases in aging patients: our preliminary experience. *Digestive Diseases and Sciences*.

[8] Qian Y., Bai T., Li J. (2018). Magnetic-guided capsule endoscopy in the diagnosis of gastrointestinal diseases in minors. *Gastroenterology Research and Practice*.

[9] Ching H. L., Hale M. F., Sidhu R., Beg S., Ragunath K., McAlindon M. E. (2019). Magnetically assisted capsule endoscopy in suspected acute upper GI bleeding versus esophagogastroduodenoscopy in detecting focal lesions. *Gastrointestinal Endoscopy*.

[10] Zhao A. J., Qian Y. Y., Sun H. (2018). Screening for gastric cancer with magnetically controlled capsule gastroscopy in asymptomatic individuals. *Gastrointestinal Endoscopy*.

[11] Liao Z., Hou X., Lin-Hu E. Q. (2016). Accuracy of magnetically controlled capsule endoscopy, compared with conventional gastroscopy, in detection of gastric diseases. *Clinical Gastroenterology and Hepatology*.

[12] Zou W. B., Hou X. H., Xin L., Liu J., Bo L. M., Yu G. Y. (2015). Magnetic-controlled capsule endoscopy vs. gastroscopy for gastric diseases: a two-center self-controlled comparative trial. *Endoscopy*.

[13] Liao Z., Duan X. D., Xin L. (2012). Feasibility and safety of magnetic-controlled capsule endoscopy system in examination of human stomach: a pilot study in healthy volunteers. *J Interv Gastroenterol.*.

[14] Keller J., Fibbe C., Volke F. (2011). Inspection of the human stomach using remote-controlled capsule endoscopy: a feasibility study in healthy volunteers (with videos). *Gastrointestinal Endoscopy*.

[15] Qian Y., Wu S., Wang Q. (2016). Combination of five body positions can effectively improve the rate of gastric mucosa’s complete visualization by applying magnetic-guided capsule endoscopy. *Gastroenterology Research and Practice*.

[16] Zhu S. G., Qian Y. Y., Tang X. Y. (2018). Gastric preparation for magnetically controlled capsule endoscopy: a prospective, randomized single-blinded controlled trial. *Digestive and Liver Disease*.

[17] Rey J. F., Ogata H., Hosoe N. (2010). Feasibility of stomach exploration with a guided capsule endoscope. *Endoscopy*.

[18] Bruno M., Marengo A., Elia C. (2015). Antiplatelet and anticoagulant drugs management before gastrointestinal endoscopy: do clinicians adhere to current guidelines?. *Digestive and Liver Disease*.

[19] Boustiere C., Veitch A., Vanbiervliet G. (2011). Endoscopy and antiplatelet agents. European Society of Gastrointestinal Endoscopy (ESGE) guideline. *Endoscopy*.

[20] Anderson M. A., Ben-Menachem T., Gan S. I. (2009). Management of antithrombotic agents for endoscopic procedures. *Gastrointestinal Endoscopy*.

[21] Veitch A. M., Baglin T. P., Gershlick A. H., Harnden S. M., Tighe R., Cairns S. (2008). Guidelines for the management of anticoagulant and antiplatelet therapy in patients undergoing endoscopic procedures. *Gut*.

[22] Shafer A., Doze V. A., Shafer S. L., White P. F. (1988). Pharmacokinetics and pharmacodynamics of propofol infusions during general anesthesia. *Anesthesiology*.

[23] Stark R. D., Binks S. M., Dutka V. N., O'Connor K. M., Arnstein M. J., Glen J. B. (1985). A review of the safety and tolerance of propofol (‘Diprivan’). *Postgraduate Medical Journal*.

[24] Chen Y. Z., Pan J., Luo Y. Y. (2019). Detachable string magnetically controlled capsule endoscopy for complete viewing of the esophagus and stomach. *Endoscopy*.

[25] Ford A. C., Marwaha A., Sood R., Moayyedi P. (2015). Global prevalence of, and risk factors for, uninvestigated dyspepsia: a meta-analysis. *Gut*.

[26] Ford A. C., Marwaha A., Lim A., Moayyedi P. (2010). What is the prevalence of clinically significant endoscopic findings in subjects with dyspepsia? Systematic review and meta-analysis. *Clinical Gastroenterology and Hepatology*.

[27] Marya N. B., Jawaid S., Foley A. (2019). A randomized controlled trial comparing efficacy of early video capsule endoscopy with standard of care in the approach to nonhematemesis GI bleeding (with videos). *Gastrointestinal Endoscopy*.

